# 852. Multimodal Deep Learning with Feature-level Fusion for Inhalation Injury Severity Prediction in Burn Patients

**DOI:** 10.1093/jbcr/irag033.322

**Published:** 2026-04-06

**Authors:** Chih-Jung Huang, Wei-Chun Chen, Ying-Jia Lin, Cheng-I Yen

**Affiliations:** Chang Gung Memorial Hospital, New Taipei City, New Taipei; National Yang Ming Chiao Tung University, Taoyuan City, Taoyuan; Chang Gung University, Taoyuan City, Taoyuan; Chang Gung Memorial Hospital, New Taipei City, New Taipei

## Abstract

**Introduction:**

Inhalation injury is a key predictor of morbidity and mortality in burn patients, often requiring early airway protection. Bronchoscopy, while considered the diagnostic gold standard, is invasive, resource-intensive, and frequently unavailable in emergency settings. As a result, clinical decisions often rely on subjective signs—such as facial burns or carbonaceous sputum—which lack reliability and can lead to misclassification. Furthermore, early physiological indicators may be masked by prehospital interventions. These limitations highlight the need for a non-invasive, objective, and real-time decision support tool. Recent advances in artificial intelligence and multimodal learning offer promising opportunities to integrate clinical and imaging data for more accurate severity prediction.

**Methods:**

We developed a multimodal deep learning framework that integrates tabular clinical data and facial photographs to predict inhalation injury severity. A two-stage architecture was implemented: separate unimodal encoders—a ResNet-50 for image data and a multilayer perceptron (MLP) for 13 SHAP-selected clinical features—were trained independently. Their hidden representations were concatenated and fed into a support vector machine (SVM) classifier to enable feature-level fusion. The dataset included 225 burn patients admitted to a tertiary burn center between 2008 and 2024, annotated by bronchoscopy (grades 0–1: n = 155; grades 2–4: n = 70).

**Results:**

Unimodal classifiers achieved moderate performance (best tabular F1-score: 87.8%; best image F1-score: 54.2%). In contrast, the multimodal SVM classifier substantially outperformed both baselines (F1: 95.7% for mild, 91.0% for severe). An ablation study confirmed the additive value of each modality and the effectiveness of feature selection. Latent space visualizations and error-overlap analyses revealed that multimodal fusion significantly reduced false negatives in severe cases by correcting misclassifications from both unimodal branches.

**Conclusions:**

This two-stage multimodal fusion framework offers a non-invasive, accurate, and scalable alternative to bronchoscopy for inhalation injury stratification. It enables earlier identification of high-risk patients, supports timely intubation alerts, and optimizes emergency resource utilization. Our findings highlight the clinical utility of integrating AI-based decision support into acute burn care workflows.

**Applicability of Research to Practice:**

This research provides a non-invasive, AI-driven tool to assist in early identification of severe inhalation injury in burn patients. By enabling earlier airway intervention and reducing unnecessary bronchoscopy, it can improve patient outcomes and optimize critical care resource allocation in emergency settings.

**Funding for the study:**

N/A.

